# PROTOCOL: The effects of road infrastructure, and transport and logistics services interventions on women's participation in informal and formal labour markets in low‐ and middle‐income countries: a systematic review

**DOI:** 10.1002/CL2.200

**Published:** 2018-09-28

**Authors:** Manisha Gupta, Souvik Bandyopadhyay, Meerambika Mahapatro, Shreya Jha

## Background

### The problem, condition or issue

Mobility is one of the key dimensions of gender equality. Helping women to carry out their household responsibilities, mobility also provides access to education, income opportunities, and to other social and economic resources. There is no dearth of documentation attesting to the fact that women's travel needs and patterns are different from those of men (World Bank, 2012; Babinard, 2011; Peters, 2001). Also, it is well evidenced that women make more trips, using a greater variety of routes, but within a more restricted geographical area, and while utilizing less expensive modes of transport than do men (ITF, 2011; World Bank, 2011; Peters, 2001). However, women from developing countries, in particular, have to balance their productive, social, and reproductive roles with poor or no access to transport, mobility related social constraints, and, often, fall under a lower income threshold (Fizzah, 2017; World Bank, 2012; Kunieda & Gautheir, 2007).

In developing countries, rural women travel mostly on foot, making several short‐distance trips around the household vicinity. These trips have to do with women spending a great deal of time on water and firewood portage taking care of their daily household consumption needs. Again, they also travel on foot to their informal, non‐farm and/or agricultural employment place, visiting the markets for crop marketing, or taking to the fields for crop harvesting. Often, women and children carry loads on their head or back as a means of freight transport, mainly of agricultural production or fodder. Therefore, the act of walking, and in some contexts, that of walking while carrying loads on the head, or the use of intermediate means of transport, such as donkey‐pulled/bullock‐pulled carts and bicycle‐on‐wheels, remain the primary mode of travel (Srinivasan, 2002, Venter et al., 2006; cited in Porter, 2008; Fernando & Porter, 2002). According to survey data collected from the Mekong Delta in Vietnam, women tend to walk and use non‐motorized transport more than do men, so that women take longer travel times than do men for same distance travelled, even though men travel longer distances than do women in same time. Expectedly, while using public transport, women travel more often in off‐peak hours, undertaking a number of tasks in connected trips (World Bank, 2011).

In the urban areas in developing countries, it is noteworthy that working women have higher demands on their time because of balancing their dual responsibilities, mainly, between economic and household activities. Again, walking is the most commonly used mode of travel for women (in Africa, for example, 57 per cent in Bamako, 69 per cent in Niamey and 73 per cent in Dakar travel on foot). In addition, a lack of access to transport or a lengthy commute can pull back women from taking up formal or higher‐paying jobs, largely because of inability to keep up to her job and household responsibilities after making long distance commutes from home (ITF, 2011). A study in Jakarta found a decline in the number of women who commute after the age of twenty‐nine years, probably because of the inability to negotiate the challenge of balancing household and work responsibilities (Rachmad et al., 2012).

The big concerns for women with low mobility are issues, such as, the safeguarding of personal safety, protection from physical harassment on public transport, and high transport cost. For instance, the lack of sidewalks, and/or stabilized hard shoulders separated from road pavements make walking difficult and unsafe for women. Further, traveling on public transport, both formal and informal, is not often the most easiest as women often encounter problems in the form of sexual harassment in overcrowded minibuses or buses. In certain geographic regions, cultural factors take the form of problematic issues preventing women from gaining access to intermediate transport systems (rickshaws, handcarts, animal‐drawn carts, bicycles, and mopeds); and, from using formal public transport without being escorted by a male family member. In addition, high transport cost prohibits women with low income to access private, public, and intermediary means of transport.

In the labour markets in low‐ and middle‐income countries (LMIC), the majority of work done by the women's labour force falls under the “vulnerable work” category (Kabeer, 2008). In informal cross‐border trade, the women traders use road transport to reach their products to the market, but the lack of or poor condition of road infrastructure translates into delays, missed market days, and perished goods (UNECA, 2010, p. 466). As far as women's small share in formal enterprises is concerned, having limited mobility due to poor transport infrastructure is one of the constraint, in addition to other gender‐related social constraints, including theirs demonstrating a low likelihood to access formal banking services, such as debt and equity financing; a lack of access to land and property (including individual rights, joint tilting, and group rights); an expensive and long enduring business registration process (UN, 2009).

Thus, the main barriers impacting women's decision to travel are high transport costs, lack of access to transport, time spent in long distance commutes, social and religious norms, and security constraints (Babinard, 2011). Gender‐related infrastructural constraints are a significant aspect of disadvantages women traders face within the informal and formal labour markets due to restricted access to markets, preventing a safe and time‐efficient mobility for women (Winters et al., 2008). The lack of transport infrastructure—particularly, road and transport services networks for accessing education and job markets—is a critical factor in women's decision not to take up formal, well‐paid labour opportunities with contractual wages and decent working conditions (Barrientos, 2002; cited in Kabeer, 2012).

According to a UN (2009) report, improvement in transport infrastructure can significantly reduce women's ‘time‐poverty’ in rural areas, as well as increase their access to markets, schools, and services, with cascading effects on women's productivity, health, and well‐being (p. 8). Transport‐driven access to basic utility services and markets have the potential to improve women's wellbeing by decreasing the amount of time spent on domestic tasks, and by a concurrent release of time for income generation (World Bank, 2012; Malmberg, 1994). Research evidence shows that transport infrastructure interventions and policy programmes with conducive gender dimensions have the potential to provide equal access to economic resources; and/or, to remove structural gender inequalities from the labour market (World Bank, 2012; Sicat, 2007; Jahan & McCleery, 2005).

In the past decades, there has been a significant investment in the gender‐responsive road infrastructure‐related transport and logistics services. The transport infrastructure solutions for safe and secure border crossing are being integrated into transport designs. Besides, innovative approaches are being taken in order to integrate gender concerns in specific aspects of road construction projects, such as involving women in different phases of construction from consultation, to appraisal, to training for roadside landscape, to the imparting of training to women as road maintenance contractors. Some of the women's empowerment imperatives implemented as gender‐responsive interventions in back‐end logistics initiatives include giving them training in customs and border requirements, and processes; and, advertising of customs and border requirements, and, costs.

This review will attempt to collect evidence that attest to the effectiveness of gender‐responsive road infrastructure, and transport & logistics services interventions on women's participation in informal and formal labour markets.

### The intervention

The review will include gender‐responsive road infrastructure, and transport & logistics services interventions, which typically include setting up of trade corridors, development of feeder road networks, arranging for private and public transport services, provisioning for safety and security, and providing customs and border management for women.

A gender‐responsive policy or programme weighs in gender norms, roles, and forms of inequality, with measures taken to actively reduce their harmful effects (WHO, 2011). Again, the term gender‐responsive is closely associated to that of gender sensitive. Precisely, the term gender sensitive takes into account the impact of policies, projects, and programmes on men, women, boys, and girls in an attempt to mitigate the negative consequences of women‐unfriendly policies and programs. While engendering is to make the process or activity gender‐sensitive or gender‐responsive by incorporating gender needs and interests, and/or eliminating gender discriminatory policies, strategies and practices, gender mainstreaming is a strategy for making women's as well as men's concerns and experiences an integral dimension of the design, implementation, monitoring, and evaluation of policies and programmes in all their spheres of political, economic and societal ramifications (WHO, 2011).

In this review, gender‐responsive road infrastructure, and transport & logistics services interventions are those interventions in urban and rural settings that incorporate women's mobility needs, provide access to safe, secure, and affordable transport, connect transport services to health care, educational centers, and markets. Examples of interventions include the development/upgrading of feeder roads and regional trade corridors, the provisioning of gender‐responsive adequate service routes and schedules, the charting of affordable fares, the implementation of safety on public transport fleet and facilities, the formulating of legal and policy provisions for gender‐responsive road sector programmes and projects, the undertaking of training and education on gender sensitivity in the transport sector, and the liberalizing of the provision of transport services that will lower transport costs to women's microenterprises (such as community‐based credit schemes) to help increase the use of intermediate means of transport (IMT). For example, giving away bicycles or financial solutions, like credit schemes, not only provide relief to women's affordability or purchase of IMT, but also show an increase in girls' enrollment in secondary schools in rural areas (Murlidharan, 2013).

Gender‐responsive transport infrastructure projects are designed to specifically emphasize women's participation/consultations in the planning of transport systems. This not only allows a good integration of gender concerns in the planning and design of transport systems but can also maximize the impact of the gender activities. For example, women's participation in discussions about type of road construction, where the road will be built, types and frequency of vehicles to be allowed on roads, setting up of wayside public amenities, such as, bus stops, public lavatories, and construction of footpaths, cross‐overs/underpasses, and footbridges can have a significant impact on women's motivations to access transport. Women's participation, free and informed consent of women to the relocation of their enterprises prior to relocation, access to adequate, fair compensation as a result of relocation, and their views from consultation in rural road and/or feeder road improvement projects, in which their roadside economic activity is relocated due to road improvements, is important because women's economic activities are an important source of income for households. For instance, the Mumbai Metropolitan Regional Development Authority's gender‐inclusive resettlement of Mumbai's 60,000 people ensured that the resettled women could continue their support systems and economic activities through joint tilting of rights of final resettlement properties in both the husband's and the wife's names (Mishra, 2009; cited in Babinard, 2011). Also, a gender‐responsive project design facilitates gender work during the implementation phase of road infrastructure. Such projects can range from the paving of dirt roads to facilitating two‐way traffic; the straightening or the upgrading of dual carriageway/motorways; and, the upgrading or construction of a feeder road.

Cross‐border women traders from LMICs have lower literacy levels, tend to have little knowledge about cross‐border trade regulations and procedures, and also face higher levels of corruption, harassment, and rights violations at times of border‐crossing (Brenton et al., 2011; Ndiaye, 2010; cited in Higgins, 2012, p. 2; Jones et al., 2007). Thus, trade related gender‐responsive road infrastructure, and transport & logistics services have the potential to increase women's start‐up registrations and/or business expansions. In the World Bank project titled ‘improving the conditions of cross‐border trade in Great Lake regions of Africa’, it seems that installation of lighting, surveillance cameras, and bulletin boards with official fees and tax information at Petite Barriere in Goma can address the physical safety issues of women, and may reduce bribing activities that women traders face at the border (Higgins, 2012a, p. 33). Besides, interventions such as professionalization of officials in Goma, Bakavu, and Uvira on the regulations, taxes, and fees, as well as educating the officials on human rights and gender‐based violence may improve the conduct of border officials (Higgins, 2012a, p. 33).

Often times, low‐income, semi‐urban and rural areas are poorly linked to the main transport routes and places of employment. As far as investments in rural roads are concerned, a major cause of worry is rural areas, typically, consisting of low volume traffic. Due to the lack of motorized vehicular traffic, rural road infrastructure investment yield high costs. For women, limited road network coverage means long walks to access the main arterial roads, including trunk line and feeder buses. Hence, gender‐responsive road investment interventions that provide access to health and educational facilities in the concerned geographical areas would be more suited to the needs of women.

As for safety and security concerns which have to do with the women feeling a loss of security when waiting for public‐transit vehicles on isolated routes and urban peripheral areas, including at bus stands and terminals, they are a major constraint on women's mobility for education and employment. Making efforts to provide well‐lit streets, stationing patrolling security officers on platforms and terminals, or on buses and trains, while raising public awareness on appropriate behaviour toward women in public transport would allow women to move securely and safely. In the context of social and cultural norms arising as mobility inhibiting factors in certain countries, interventions such as women‐only transport services help women to counter their difficulty in travelling alone. The overall concerted effort to reduce gender based violence against women can have a positive impact on how women use transport services. Successful examples of such interventions include, Banat taxi –pink taxis driven by women for women—in Beirut, Tehran (World Bank, 2010; cited in Babinard 2011). Likewise, women‐only light rail or metro cars have been introduced in several LMIC cities.

### How the intervention might work

According to the behavioral approach in location theory (Pred, 1969), location accessibility and economic activity are interrelated. Accessibility is the direct outcome of a transportation infrastructure, in place, to support mobility. Thus, transport infrastructure access has an indirect impact on any economic activity, such as, market expansion, cost‐effective distribution of goods, and the enabling of more people to reach workplaces or access public services. Historically, traditional transport planning models did not consider gender differences in travel activity patterns, and in particular, for those differences that arose from purpose of trip, frequency and distance of travel, and mode of transportation used. Because of a limited access to private vehicles, IMT, or public transport; not to mention, the existing gendered division of labour, transport infrastructure access did little for women in low skilled, low‐income rural/semi‐urban peripheral areas, especially in LMIC except harden the feeling of paucity of time faced from tighter time constraints compared to that experienced by their male counterparts. Therefore, gender‐responsive road infrastructure, and transport & logistics interventions facilitate women's physical mobility and reduce transportation costs, which in turn has positive effects on their economic empowerment.

**Figure 1:**
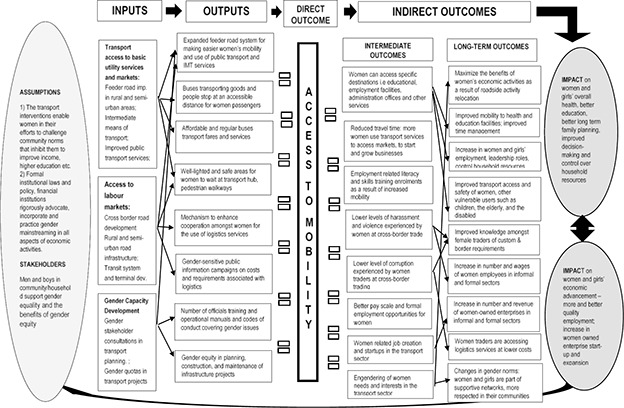
Theory of change

We have developed a theory of change of road infrastructure, and transport & logistics services interventions outlining how these interventions might work. Theory of change Figure 1 (Appendix 1) provides a framework of how road infrastructure, and transport & logistics services interventions may improve women's accessibility and long‐term outcomes, such as, women's economic advancement –participation in informal and formal labour markets.

For example, gender equity in planning, construction, and maintenance of infrastructure projects includes the promoting of women's participation in roadwork contracts, and the planning of transport systems. The act of including women in stakeholder consultations, while planning for transport systems, often provides practical insights that can improve transport access and safety of women, as well as for other vulnerable users, such as children, elderly, and disabled persons. Women's participation and their views from consultation in road improvement projects, as a result of relocation of roadside economic activities, can maximize the benefits as an outflow of their economic activities created through road improvements. With women's participation and consultation, the measures to reduce the risk of increased HIV/AIDS and human trafficking can also be addressed in the context of these projects (DANIDA, 2006).

Additionally, the theory of change indicates that improved mobility as a result of good quality transport access may lead to mutually exclusive positive impacts on (a) women and girls' overall health, education, decision‐making power, and control of household resources, and, on (b) women and girls' economic advancement, for example, their entitlement to formal and better quality employment opportunities, as well as increase in or expansion of formal women‐owned enterprise start‐ups. This can be an ongoing process, inhering in positive impacts strengthening the assumptions made, which, in return, will accelerate the number of positive outcomes and overall impact, until women's transition into the formal labour market is accomplished to its optimum level.

Cultural context and key stakeholders (community and household men) can influence the success or impede the benefits of interventions. For example, it is important that men and boys support gender equality and the benefits of economic empowerment of their mothers, wives, sisters, and daughters. If cultural practices that do not allow women's participation outside household activities are a major barrier, then accessibility will be partially effective in producing results that while improving the overall health and bettering education does not do anything for economic empowerment of women. Heterogeneity among women can influence the final outcomes of transport interventions. Women's characteristics such as education, age, income, and personality traits may play an important role, particularly in generating labor market related impacts from transport interventions. On the one hand, there could be women who are willing to transform access to mobility for better employment opportunities or start‐ups, on the other hand, there could be women for whom impacts may be minimal because they do not have the characteristics that can make them benefit from access to mobility that much. According to the study (Koolwal & Van de Walle, 2013) better access to water is not found to be associated with greater off‐farm paid work but is associated with less unpaid work for women. Additonally, some transport interventions can bring benefits in short term while other interventions can generate benefits in a longer time frame. For example, labor based road schemes can bring immediate social benefits as well as short term incomes to women. In long term, policy interventions to involve women in road maintenance enterprises can lead to transparency in local government and empower women to participate in meetings and form micro‐enterprises.

Nevertheless, the success of the theory of change relies on a number of assumptions. One of the key assumptions of the theory of change is that of transport interventions enabling or supporting women in their efforts to challenge community norms (a lack of safety in public transport, long commutes, inability to contribute to household work due to long hours away from home) which would otherwise inhibit them from attaining higher education and securing better employment/entrepreneurial activities. Another key assumption is the presence of well‐rounded legal, financial, and social institution laws and policies that support women's empowerment, for example, exercising equal rights in parental property, benefiting from workplace policies with regard to hiring of married women, and taking opportunities created for speaking up against sexual harassment of women. In the transport accessibility‐to‐market participation concerns are those of social constraints, such as, restrictions on women's movement within the neighbourhood of their home and vicinity of community, or prohibitions made by other household members against working in male‐dominated sectors, all of which may prevent women's participation in labour markets, even if they have higher educational qualifications and business skill set. Similarly, if there is neither access to nor financial support for child care, or if it may so happen that women spend too much time on domestic chores or care work, then it is likely that they will have little energy or motivation to pursue empowerment of their own status. Ultimately, even where women are able, inspite of constraints, to accomplish higher educational goals and acquire business skills, the overriding need for affordable child care facilities of those with children may affect their participation in high‐skilled occupations. Further, women microentrepreneurs require access to easy loans and business registration process, but often have no assets in their name. Notwithstanding, regulations such as equal rights in parental property being critical, workplace policies with regard to hiring of married women, opportunities created for speaking up against sexual harassment, and job security may also influence the final outcomes.

### Why it is important to do the review

Several evaluation reports have assessed some measure of women and girls' economic empowerment in one or more of the following areas: financial services, business development services, skills training, and social protection (Banerjee et al., 2013; Deininger & Liu, 2013; Jones & Shaheen, 2013; Oxfam 2013; Ahmed, et al., 2009; Valley Research Group & Mayoux 2008; Garikipati, 2008; Devereaux et al, 2007; Karlan et al, 2007); trade access to markets (Nelson & Smith 2011; World Bank & IFC 2011; Bacon 2010); and regulatory and legal frameworks (Uwayezu & Mugiraneza, 2011; World Bank & IFC 2011). Similarly, several systematic reviews have measured the effectiveness of gender‐responsive social and economic interventions on women's empowerment. There are reviews on the effectiveness of money transfers, in cash and/or in kind, as well as those on land ownership, microfinancing, and business training interventions on women's empowerment (Brody et al., 2015; Dekker, 2013; Yoong et al., 2012; Vaessen et al., 2012; Stewart et al., 2010). On road infrastructure, the impact of rural road extensions has been reviewed (Hine et al., 2016), but the review did not include sex‐disaggregated outcomes for women and girls.

Overall, the research findings demonstrate that mixed interventions (economic skills and services alongside life skills and other training services) appear to be effective from women's economic empowerment perspective (Taylor et al., 2014). On the other hand, stand‐alone interventions, such as, financial services have a positive impact on women's economic empowerment (Brody et al., 2015; Dekker, 2013; Yoong et al., 2012), but have a limited impact or no effect on female bargaining power in the household, on feminine needs‐based goods or for children‐related ones, or even female health outcomes (Taylor et al., 2014; Vaessen et al., 2012; Stewart et al., 2010).

However, there is no review, extant, that has explicitly measured the effectiveness of road infrastructure, and transport & logistics interventions, on women's use of transport, and theirs experiencing, consequently, an increase in participation in labour markets in LMIC, which thus remains unknown. This review could also provide an insight into the question if stand‐alone transport infrastructure interventions have an effect on women's bargaining power in the household.

In the 1990s, transport planners, economists, and policy makers globally, recognized the differences in travel activity‐related patterns between men and women, and that past interventions in the transport sector have not responded well to the needs of women. As a result, gender concerns began to feature in the agendas of international agencies, and in the transport policies and programmes of their member governments. In 2001, World Bank incorporated a gender mainstreaming strategy into the Bank's country diagnostic work, lending operations, and technical assistance. In 2007, the World Bank adopted the Gender Action Plan (GAP) to integrate gender concerns into operations spread across regions, particularly, in the operations within the non‐social sectors, such as, the infrastructure and the private sectors. The new World Bank gender strategy 2016‐2023 action plan aims to address challenges related to gender equality and empowerment. One of its objectives include removing constraints for more and better jobs as a result of lack of care services, unsafe transport, occupational sex segregation, and entrepreneurship (World Bank, 2017). In 2008, the Asian Development Bank (ADB) incorporated the gender equity plan in the transport sector as one of the operational priorities under its long‐term strategic framework, Strategy 2020. The UNECE Inland Transport Committee included transport and gender issues in its agenda for the first time in February 2009.

As a result, significant investments in gender‐responsive transport operations have been made, with spillovers strengthening women's empowerment in its other social dimensions (uplifting poverty, and rural development are some worth mentioning). Some examples include women's participation in construction of rural road projects (Peru, Lesotho, Yemen and many others in South and East Asia, and Sub‐Saharan Africa); access to markets by promoting intermediate means of transport (Madagascar, Tanzania); improving urban road safety and security by consulting women in proposed design features (China); incorporating gender into the community road safety project (Bangladesh); gender sensitive port and railway restructuring ‐ (Mozambique, Zambia, Macedonia); and, policy study on rural access and mobility with special focus on gender (Pakistan).

From our preliminary searches, we have identified some nonrandomized studies in LMIC that assessed the effect of gender‐responsive road infrastructure, and transport & logistics services interventions. Khandker et al. (2006) examined the impacts of the two rural road‐paving projects in Bangladesh by using a quasi‐experimental household panel data set and control villages before and after program implementation. The rural road investments have been found to reduce poverty through higher agricultural production, lower input and transportation costs, and higher employment for men and women. Gomez et al. (2011) explored the effect of the expansion of cross‐border roads and bridges between Laos and Thailand on traders' activities, highlighting changes effected in gender roles and perceptions acquired of entrepreneurial activity taking off from women's participation, in two research sites. In Morocco, a rural road program intervention led to an increase in the enrollment of girls in schools (Levy, 2004). Yamauchi et al. (2011) examined the impact of spatial connectivity on household income growth and nonagriculture labour supply in Indonesia. The empirical results showed that the post‐primary education scene is witness to significant increases in the benefit arising from the improvement of local spatial connectivity in remote areas by promoting labour transition to nonagricultural sectors. Table 1 shows a list of primary studies on gender‐responsive road infrastructure and logistics interventions (Table 1).

**Table 1:** List of primary studies and systematic reviews on gender‐responsive road infrastructure and logistics interventions

**Authors**	**Title of the study**	**Study**
Chowdhury S. 2010	Impact of infrastructures on paid work opportunities and unpaid work burdens on rural women in Bangladesh. *Journal of International Development, 22* (7), 2010.	Economic Modeling study
Gomez, Jr., José Edgardo, Nittana Southiseng, et al., 2011	Reaching across the Mekong: local socioeconomic and gender effects of Lao‐Thai cross‐border linkages. *Journal of Current Southeast Asian Affairs, 30* (3), 3‐25, 2011.	Cross‐sectional study
Khin Hnin Phyu	Negotiating the Trade Route at the Border: A Case Study of Women Small‐Scale Cross‐Border Traders in Myitkyina, Myanmar. In K. Kusakabe (Ed.) *Gender, Roads and Mobility in Asia*. London: Practical Action Publishing.	Case study
Mahapa S. and Mashiri M. 2001	Social exclusion and rural transport: Gender aspects of a road improvement project in Tshitwe Northern Province. *Development Southern Africa, V 18* (3), 2001.	Observational study
Maertens, M., & Swinnen J.F.M. 2008	*Gender and modern supply chains in developing countries* (LICOS Discussion Paper 231). Leuven: LICOS Centre for Institutions and Economic Performance, 2008.	
Murlidharan K., & Parekh N. 2013	*Cycling to School: Increasing Secondary School Enrolment for Girls in India* (NBER Working Paper No. 19305), 2013.	
Ogbonna, M. O. and Nwaobiala, C. U. 2014	Effect of Fadama III Project on Rural Farm Women Production In Gombe State, Nigeria. *Nigerian Journal of Agriculture, Food and Environment, 10(1), 13‐18, 2014.*	Controlled before and after
Shahidur R. Khandker, Zaid Bakht, Gayatri B. Koolwal 2009	The Poverty Impact of Rural Roads: Evidence from Bangladesh. *Economic Development and Cultural Change,* 57 (4), 685‐722, 2009.	Quasi‐experimental study
Thammanosouth, Saykham, Viengnam Douangphachanh, and Lamphoun hounphakd	Gender Analysis of Changes in Livelihoods at the Case study Border: A Case Study of Houayxay, Lao PDR. In K. Kusakabe (ed.) *Gender, Roads and Mobility in Asia*. London: Practical Action Publishing.	Case study
Win Myo Thu	Impact of Cross‐Border Road Construction on the Livelihoods of Women and Men in Kyaing Tong Tachilek, Myanmar. In K. Kusakabe (ed.) *Gender, Roads and Mobility in Asia*. London: Practical Action Publishing.	Case study
Yamauchi, F., Muto, M., Chowdhury, S., Dewina, R., Sumaryanto, S. 2011	Are schooling and roads complementary? Evidence from income dynamics in rural Indonesia. World Development, Vol 39 (12), 2232–2244, 2011.	
**Reviews**		
Gupta Manisha, Menon Geetha, Devkar Ganesh, and Thomson Hilary 2016	Regulatory and road engineering interventions for preventing road traffic injuries and fatalities among vulnerable road users in low and middle income countries.*DFID ‐ Cochrane Public Health Group Systematic Review (ongoing),* 2016.	Ongoing
Hine, J., Abedin, M., Stevens, R.J., Airey, T., Anderson, T. 2016	Does the extension of the rural road network have a positive impact on poverty reduction and resilience for the rural areas served? If so how, and if not why not? A systematic review. London: EPPI‐Centre, Social Science Research Unit, UCL Institute of Education, University College London, 2016.	Review
Yoong J., Rabinovich L., Diepeveen S. 2012	The impact of economic resource transfers to women versus men: a systematic review (Technical report). London: EPPI‐Centre, Social Science Research Unit, Institute of Education, University of London. 2012.	Review

This review will address a topic of particular relevance in tackling gender inequalities and how road transport infrastructure can enhance women's economic empowerment in informal and formal labour markets in LMICs. The findings of this review will provide valuable and contextually relevant data for what works, and what does not, in gender‐responsive interventions in the transport sector. The findings of this review will have an important implication in the transport infrastructure policy designing and the making of provisions for gender equity.

## Objectives

The primary objective of this review is to measure the impact of gender‐responsive road infrastructure, and transport & logistics interventions. The secondary objective is to examine the factors that constraint (or drive) the empowerment of women as a result of gender‐responsive road infrastructure, and transport & logistics services interventions.

This review will aim to answer the following research questions:

1) What is the effect of road infrastructure, and transport & logistics services interventions on women's participation in informal and formal labour markets as wage employees and as self‐employed (owners of start‐ups/businesses) in low‐ and middle‐income countries?
2) What are the factors that constraint (or drive) the empowerment of women as a result of gender‐responsive road infrastructure, and transport & logistics services interventions?

## Methodology

### Criteria for including and excluding studies

Due to limited evidence availability, we will apply broad study inclusion criteria, nevertheless, we will critically appraise included studies. Studies will be synthesized by risk of bias status. Before making decisions about which studies should be included in the meta‐analysis, a sensitivity analysis will be conducted to examine variation in reported effects.

#### Types of study designs

To answer our review question, we will include quantitative and qualitative study designs for evidence and analysis appropriate to each review objectives.

**Review question 1:**

Although randomized controlled trials (RCTs) will be the preferred study design, preliminary literature review has indicated a low possibility of detecting such study designs in the transport sector. The review will include the randomized control trials and nonrandomized studies. We will include the following study designs:

- Randomized controlled trials: randomization can occur at the individual or cluster level. Eligible comparison conditions include inactive control groups or business as usual.

Nonrandomized studies:

- Controlled before and after: In this study design, changes in key outcomes between the before‐intervention and the after‐intervention periods will be assessed for the intervention and control group. Studies must have used appropriate methods to control for selection and confounding, such as statistical matching (propensity score matching or covariate matching), regression adjustment (difference‐indifference, fixed effects regression, single difference regression analysis, instrumental variables, and ‘Heckman’ selection models). The time interval for before and after period may be continuous (without a time interval) or comparative (a time interval exists for each of before‐intervention and after‐intervention periods). Comparison conditions include no comparison or “business as usual.”
- Uncontrolled before and after: This study design rely on cross‐sectional data to compare changes in outcomes at a before‐intervention and an after‐intervention implementation. There is no control group, often participants act as their own control. The time interval for before‐intervention and after‐intervention periods may be continuous (that is, they are without a time interval) or comparative (a time interval between the before and after time period). Comparison conditions include no comparison or “business as usual.”
- Regression discontinuity design: The regression discontinuity design is a pretest‐posttest comparison group design. The participants are assigned to the programme or comparison groups solely on the basis of a cutoff score on a pre‐program measure.
- Analytical cross‐sectional studies with comparison/control: analytical cross‐sectional studies with comparison or control measure both the exposure and outcome of interest at a point in time for the purpose of comparing outcome differences between exposed and non‐exposed, for example comparing places with different degrees of access or road infrastructure conditions.

The study designs, such as uncontrolled before and after are considered less suitable for inclusion in systematic reviews because of the high risk of biases and low quality evidence; however, previous Cochrane Reviews (Bunn et al., 2003; Thomson et al., 2013; Gupta et al., 2015 [ongoing] have included such study designs. In the review on transport infrastructure for vulnerable road users' safety in LMIC (Gupta et al., 2016), of the 18 studies that matched eligibility criteria, 1 CBA, 5 ITS (high quality study designs), and 12 uncontrolled before‐ and after‐intervention studies were identified. We think that the systematic evaluation of what is expected to be a considerable amount of research is crucial mainly because without collecting and critically analysing these studies, they are currently disregarded. There might be significant unknown effects that can be valuable for the stakeholders regardless of whether the evidence is of low quality. A recent Cochrane review of reviews has shown that there is insufficient evidence of significant effect estimate differences between RCTs and observational studies (Anglemyer, 2014).

We will include studies that measure change in women's economic advancement individually, within the household, and in the community. Other inclusion criteria include intervention implementation and data collection at individual level, group level, or both.

**Review question 2:**

The review will include qualitative studies that use qualitative methods for data collection and analysis and mixed methods studies that use quantitative and qualitative methods for data collection and analysis:

- Descriptive studies (surveys) collect data at one particular point in time for all participants using quantitative data collection methods. Descriptive studies report descriptive statistics and observations such as opinions, behaviours, and perceptions
- Qualitative studies using in‐depth interviews or focus groups: In‐depth interviews are structured or semi‐structured face‐to‐face discussions between the researchers and participants. In general, they explore in detail the participant's experiences and attitudes regarding a particular occurrence/issue. Focus groups involve groups of participants (usually 12 to 15 participants) who are bought together to participate in guided discussions about a specific topic or issue.
- Case studies/series involve an in‐depth examination of a subject of study (the case/cases), as well as its related contextual conditions. For example, women's views and experiences of a particular issue, problem, or intervention.
- Impact evaluation reports included to address research question 1, provided they contain qualitative or descriptive information relevant to addressing research question 2.
- Process evaluations determine whether the intervention/program activity has been implemented as intended. Process evaluations may collect qualitative and quantitative data from different stakeholders to cover subjective issues, such as perceptions of intervention success or more objective issues, such as how an intervention was operationalized (Snilstveit et al., 2015).

To be included, studies must adequately report (1) aims of the research; (2) research methodology; (3) how gender insensitive transport affects exclusion; (4) how gender‐responsive transport affects inclusion; (5) discussion of factors that constraint (or drive) women's outcomes due to low or no access to road infrastructure, and transport & logistics services interventions.

The data from included studies will be used for (1) situation/context analysis, (2) establishing why change should takes place or did not take place, (3) researching how and if women overcome transport constraints, (4) if not, what were the reasons.

We will exclude feasibility and modelling studies and certain regulatory studies, such as, the conduct of women drivers, conductors, and passengers, as well as the conduct of border custom staff and management with regard to women's migration, women's human rights, and sexual harassment. In order to keep the scope of the review manageable, we will not include project implementation studies.

#### Types of participants

Women of all ages 16+ living in low‐ and middle‐income countries, as classified by the World Bank list of economies.

#### Types of interventions

The review will include road infrastructure, and transport & logistics services interventions, under the broad categories listed below. This is not an exhaustive list, a combination of one or more of these interventions or with other approaches could also be considered:

**I Transport access to basic utility services and markets**

**1.1 Feeder road improvements in rural and semi‐urban areas**

Many rural women who lack access to motorized transport tend to travel on feeder roads and will use intermediate means of transport, such as, donkey carts, bicycles, and motorcycle taxis to access markets, health, and education centers. Gender‐responsive interventions include repair of potholes, installation of traffic lights, construction/improvements of footpaths, foot‐bridges, exclusive sidewalks for women.

**1.2 Intermediate Means of Transport (IMT)**

Intermediate means of transport are less expensive modes of transport, that include bicycles, motorized two‐wheelers and three‐wheelers, tractors, farm trucks, and various animal‐drawn carts and wagons. IMTs can play a vital role in the access to markets, schools, and health care facilities in rural and semi‐urban areas. Women find it much more difficult to get a profitable paying return in using IMTs because of the high maintenance costs. IMT interventions include:

1.2.1 Bicycle programs for women and girls in rural areas.

1.2.2 Community‐based financial solutions and/or credit schemes to help women purchase IMTs.

**1.3 Public transport service in urban areas**

1.3.1 Gender‐responsive public transport services and routes: interventions include increase in timeliness and frequency of buses and taxis, affordable transport service fare options during the off‐peak hours, access to non‐commuter or decentralized services to help women access specific destinations such as markets, educational and employment facilities, and administrative offices.

1.3.2 Safe access to public transport: one of the key factors that constrains women travel patterns and use of public transport is personal safety and security risks at transportation facilities, such as, parking lots, buses and bus stops. Safety design interventions include construction of footpaths/pedestrian walkways in urban and rural areas, well‐lit transit stops, installation of roadside lights, women‐only services at subways or women‐only train cars on commuter trains, surveillance cameras, panic/alarm buttons, and uniformed and non‐uniformed officers who patrol public buses and bus stops.

1.3.3 Affordable public transport fares: women in urban as well as rural areas tend to be at the lower end of the transport expense curve. Women's travel pattern of multiple short distance trips to attend to various household duties often creates an extra transport cost burden. Interventions, such as, concessions in public bus services, use of or increase in subsidies in order to reduce fares or the making of provisions for integrated fares are helpful.

**II Transport access to labour markets**

These include: (1) rural and semi‐urban road infrastructure improvements; (2) cross‐border road development; (3) logistics interventions at cross‐border trade.

**2.1 Rural and semi‐urban road infrastructure improvements**

These include economic development related road improvement interventions. Improvements in rural and semi‐urban road infrastructure can improve women's participation in economic activities. Such interventions include feeder road development, rural road fittings, like bridges and culverts along the roads, inter‐city route development, and bathroom facilities.

**2.2 Cross‐border road development**

Cross‐border roads provide the population of border towns dotting the neighboring country's borders to access the markets through informal cross‐border trading activities. Development of feeder roads connecting remote areas with main, larger roads enable women traders to connect to bigger markets. For example, the cross‐border road development in the Greater Mekong Subregion (GMS) constitutes 20,132 kilometers of road network across Cambodia, the Lao People's Democratic Republic (PDR), Myanmar, Vietnam, and Thailand. National Road Number 3 (NR3) links Thailand and PRC via the Lao PDR; Kyaing Tong‐Tachileik road links Shan State (northeastern Myanmar), Mae Sai (Thailand), and a portion of Asian Highway Network 2 (AH2); and Myitkyina‐Tengchong road runs between Myitkyina (Myanmar) and Jiego (PRC).

**2.3 Logistics services at cross‐border trade**

2.3.1 Information on informal traders' rights and obligations at border crossings, such as, posting of official fees and tax information on bulletin boards at border crossings.

2.3.2 Establishment of one‐stop windows/fast‐track clearance systems for informal cross‐border women traders.

2.3.3 Women's security at border crossing and at transport hubs.

2.3.4 Gender sensitivity training for customs and border management officials (including management).

**III Gender capacity building in transport sector**

Such interventions refer to national‐level gender‐responsive transport infrastructure projects, typically, funded by international agencies, such as, World Bank, ADB, and African Development Bank. The projects are designed to specifically include socially and culturally acceptable interventions so that traditionally excluded and disadvantaged groups, such as, women and the poor, would be key agents and beneficiaries. They emphasize women's participation in grassroots decision‐making processes and structures, employment opportunities, and ways to mitigate project‐specific social and gender‐responsive risks.

**3.1** Gender stakeholders/consultations in the planning of transport systems: A good integration of gender concerns in the planning and the design of transport systems can maximize the impact of the gender activities. Such projects can range from gender considerations in the development and the maintenance of district and community access roads to the straightening, or the upgrading to dual carriageway or motorway status; women's participation and consultation in rural road and/or feeder road improvement projects in which roadside economic activity is relocated due to road improvements. Also, inclusion of surveys, needs assessments, feedback and learnings from previous projects and countries into the design of transport infrastructure projects.

**3.2** Gender quotas in transport appraisal, procurement, and road maintenance microenterprises: These projects include promoting women in road work contracts, such as, road maintenance and landscaping. Uganda's Road Sector Development Programme (RSDP) supported by Danida's Road Sector Support Programme was designed with an objective of empowering women by improving their reach of opportunities to participate in and benefit from the roads' subsector. Timor‐Leste Road Sector Improvement project's gender‐focused initiative includes participatory and gender‐inclusive identification and selection of 46 rural feeder roads to be rehabilitated as a mode for designing socially inclusive and gender‐responsive transport projects.

**3.3** Gender sensitive curriculum and education material for transport sector staff and workers. An example of gender‐aware project design is World Bank's Anchoring Gender in Transport Projects in Rural Peru. Another instance of such a project is the ADB‐supported Third Rural Road Infrastructure Project (RDP 21) in Bangladesh that aims to transcend socio‐cultural barriers that limit economic and social participation of women by arranging for the provisioning of simple infrastructure that will increase women's visibility and inclusion in economic activities.

The review will include road infrastructure, and transport & logistics service interventions exclusively targeting women, as well as interventions targeting both women and men, provided the studies separately analyze the outcomes for women. Studies in which outcome impacts are not disaggregated by gender will be excluded.

Comparisons: Comparison of intervention versus no intervention or “business as usual.” Studies that will be included to address the research objective 2, a comparison condition, is also no intervention or “business as usual.”

We will exclude conditional cash or in‐cash transfers to women's microenterprises for other market activities (for example, direct financial cash grants or subsidies for land leases), or alternatively, programs such as the voucher/coupon‐based inputs for assets program (e.g., poultry supply, livestock, seeds, or other farm inputs), microfinancing of self‐help groups, cash or in‐cash transfers associated with improvements in household income and savings, and households' ability to withstand economic shocks.

We will also exclude trade infrastructure interventions. Because the focus of this review is to see the effects of transportation related interventions on women's economic empowerment in informal and formal labour markets in LMIC setting, we are interested only in road infrastructure and logistics services interventions that facilitate women traders' movement of goods in informal cross‐border trade.

Transport infrastructure also comprises of ports and railways sectors. Port and railway efficiency interventions will not be included.

We will not include use of technology interventions, such as, governmental efforts to use technology (e.g., mobile phones and computers) to disseminate information on market prices and logistics costs.

#### Types of outcome measures

**Review question 1:**

We will include any of the listed primary outcomes as criteria for including studies:

Primary outcomes

(1) Women's employment, including employment generated by any firm in the sectors not traditionally employing women or those in the sectors traditionally employing women. (2) Investment in productive assets, such as, land and livestock, micro‐startups including ownership and control in sectors not traditionally for women entrepreneurs or sectors traditionally for women entrepreneurs. (3) Women's earnings (wages or salaries/business revenues) in the sectors not traditionally employing women or that earned after moving sectors. (4) Informal borrowing by women.

Secondary outcomes

(1) Use of public transport services by women by number of trips made. (2) Transport related expenses by women. (3) Provision of buses/taxis/chaperons by private firms. (4) Frequency of buses and taxis services during the off‐peak hours. (5) Number of bicycle programs for women and girls in rural areas. (6) Number of women micro‐enterprises in road work/ transport infrastructure contracts. (7) Number of transport infrastructure projects as a result of women's needs and preferences/consultations.

**Review question 2:**

We will include descriptive information/narrations on the attitudes, views, experiences, and behaviours of women participants under the following sub‐categories. This is not an exhaustive list:

(1) Economic: such as educational and economic opportunities, cost of public transport or IMT, educational access and aspirations, willingness to take a job, long distance travel to markets, educational, and health centers, ‘Time poverty’;
(2) Legal‐institutional: such as considerations for women's needs in transport infrastructure, women's participation in transport sector, safe access to public transport, public information on logistics, conduct of border and custom management staff;
(3) Psychological: such as feeling of economic power/independence; autonomy/self‐confidence/self‐esteem; feeling of sexual harassment in using public transport; feeling of freedom of movement/mobility;
(4) Social/gender norms: such as women and girls are expected to do domestic work, social sanctions such as in certain countries girl must be accompanied with a man while traveling;
(5) Cultural values and practices: such as a regular pattern of behaviour like sexual harassment in public transport, male dominated transport sector jobs.

Unintended effects

There has been some evidence of positive associations between women's empowerment and lower fertility, longer birth intervals, and lower rates of unintended pregnancies; between empowerment and women's expectations or aspirations for higher studies for their daughters and subsequent delayed marriages; and, outside empowerment, it was found that women's economic independence is associated with reduced HIV risk behavior as a result of their influence in relationships; and, women's access to home‐based work, which may not have been there earlier.

Adverse effects

Women's participation in household decision‐making as a result of increased autonomy and self‐confidence may increase tension within household and/or domestic violence, divorce rate, and infidelity among partners. Also there are chances of increase in accidents, congestion, and pollution as a result of extra traffic travelling from the feeder roads to nearby towns.

#### Duration of follow‐up

We will include any follow‐up period for included studies.

#### Types of settings

We will include studies of interventions only from low‐ and middle‐income countries, and exclude studies of interventions from that of high‐income countries.

### Search strategy

We will conduct a comprehensive search covering all relevant academic databases, Google Scholar, and websites with published and unpublished research.

**Search for Review Question 1**

Terms describing population and interventions will be combined in the final search strategy. A draft search strategy (Appendix 2) consists of the following combinations: “Population and Intervention” and “Population and Study type.” We will develop a comprehensive search strategy in consultation with our information specialist. We will also search for thesaurus/ index terms. The search would need to be adapted for each database, recognizing that key terms and search functionality will differ.

**Electronic databases**

The following subscription based databases and open access databases will be searched using a comprehensive search strategy:

**Table:** 

**Database**	**Website**
Applied Social Sciences Index and Abstracts (ASSIA – ProQuest)	http://www.proquest.com/products‐services/ASSIA‐Applied‐Social‐SciencesIndex‐and‐Abstracts.html
Econlit (Ovid)	http://www.aeaweb.org/econlit/
Emrald Insight	http://www.emeraldinsight.com/
PsycINFO (ProQuest)	
JSTOR	https://www.jstor.org/
SCOPUS	http://www.elsevier.com/online‐tools/scopus
Sociological Abstracts (ProQuest)	http://www.proquest.com/products‐services/socioabs‐set‐c.html
TRANSPORT (Ovid)	http://www.ovid.com/site/catalog/databases/157.jsp
Web of Science (SSCI & AHCI)	http://thomsonreuters.com/web‐of‐science/
Ebsco's	https://www.ebsco.com/products/research‐databases/gender‐studies‐database
Google Scholar	www.scholar.google.com

We will carry out extensive manual searches of institutional websites, international agencies databases, and impact evaluation repositories for grey literature:

**Table:** 

**Institutional websites**	
Campbell Library	https://campbellcollaboration.org/library.html
3ie Impact Evaluation Repository	http://www.3ieimpact.org/en/evidence/systematic‐reviews/
EPPI Center database	https://eppi.ioe.ac.uk/cms/Default.aspx?tabid=62
Cochrane Database of Systematic Reviews	http://www.cochranelibrary.com/cochrane‐database‐of‐systematic‐reviews/
**International agency websites**	
Asian Development Bank (ADB)	https://www.adb.org
Centre for Global Development	https://www.cgdev.org/
Department for International Development (DFID)	http://r4d.dfid.gov.uk/SystematicReviews.aspx#aSystematicReviewTop
Swiss Agency for Development and Cooperation	http://www.sida.se/English/
Inter‐American Development Bank	http://www.iadb.org/en/inter‐american‐development‐bank,2837.html
International Center for Research on Women (ICRW)	https://www.icrw.org/
International Development Research Center (IDRC)	https://www.idrc.ca/
Global Fund for Women	https://www.globalfundforwomen.org/
Oxfam	www.oxfam.org
Global Regional Health Observatory	http://www.who.int/gho/en/
United Nations Economic and Social Commission for Asia Pacific (UNESCAP)	http://www.unescap.org/
SOAS Center for Development Policy and Research	https://www.soas.ac.uk/cdpr/
BRIDGE Institute of Development Studies (IDS)	http://www.ids.ac.uk/bridge
Development Impact Evaluation (DIME)	http://www.worldbank.org/en/research/dime/research
Poverty Impact Evaluation database	http://www1.worldbank.org/prem/poverty/ie/evaluationdb.htm
World Bank Gender Impact Gateway	http://www.worldbank.org/en/topic/gender/publication/engender‐impact‐a‐gateway‐to‐gender‐related‐impact‐evaluations
Harvard Gender Action Portal	http://gap.hks.harvard.edu/
	

We will use back‐referencing from included studies as well as citation tracking to identify additional relevant studies. We will contact authors of studies which we were unable to retrieve. A record will be maintained describing the databases searched, the keywords used, and search results.

Systematic reviews will be included to identify studies for inclusion.

**Search for Review Question 2**

We will conduct an exhaustive search of all relevant studies. An exhaustive search of qualitative research can be difficult to identify using only electronic databases. For one, qualitative research may be less frequently conducted, submitted for publication and/or published in high quality journals. Secondly, the standard indexing terms for locating reports of qualitative research do not exist in the same way that they do for quantitative reports (Pearson et al. 2011). Within the time‐bound production of a policy‐relevant systematic review, Pearson et al found a priory (protocol driven), targeted, and reference‐checking search approaches to be the most effective (2011). Therefore, we will undertake a multiple search strategy:

1) Protocol driven searching will be the first starting point. We will use the electronic search strategy used to identify studies for review question 1 (Appendix 2). In order to capture both quantitative and qualitative literature relevant to research questions, we will not specify study types (Centre for Reviews and Dissemination, 2009; cited in Booth, 2016, p. 13). Using study types in the search strategy would exclude relevant qualitative studies in addition to quantitative and mixed‐methods studies that omit this information from title and abstract (Booth, 2016).
2) Targeted searching of sibling studies (Booth, 2011). Sibling studies may include qualitative research, case studies, or process evaluations associated with specific included studies. Such studies are often commissioned to explore the context surrounding an effectiveness study with the aim of documenting the process and explaining contextual factors that influence implementation and/or outcomes.
3) Reference checking of relevant literature reference list

In addition, we will hand search grey literature and conference proceeding abstracts.

#### Time‐period

We will include studies conducted from 1990 to the present. Although, the UN Commission of the Status of Women (working for the advancement of women in developing countries) was established in the 1960s, the focus on the economic and social issues of women empowerment started from 1987 as part of the follow‐up to the 3rd World Conference on Women in Nairobi.

### Criteria for determination of independent findings

We will include only one effect size per study in any single meta‐analysis. Where there are several publications reporting on the same study, we will use the numerical data from the most recent publication. In cases where more than one study uses the same data set to measure an outcome variable, we will extract the effect size from the study with the lowest risk of bias. If studies present several impact estimates for different outcome variables that measure the same construct, we will use a sample size weighted average to create a “synthetic effect size.” If studies include more than one treatment arm, we will include the effect size from the treatment arm that is most similar to the other programs that are included in the meta‐analysis, and finally if a study/studies include multiple follow‐ups, we will include the follow‐up which is most similar to the other studies that are included in the meta‐analysis.

### Assessment of risk of bias

Two independent reviewers will assess the quality of each included study using the published list of criteria for research question 1 (table 2) and research question 2 (table 3). The results of the quality appraisal will be reported in the review. We will not exclude any studies from analysis on the basis of poor quality. But, we will conduct a sensitivity analysis to examine variation in reported effects by the overall study quality.

**Table 2:** Risk of bias assessment tool

**Risk of bias assessment tool**	
Justification of use	
Which primary outcomes are measured in the study?	
Provide the authors definition of each included primary outcome	
Which secondary outcomes are measured in the study?	
Provide the authors definition of each included secondary outcome	
Describe methods of data collection	
What is the frequency of outcome data collection?	
At which level was assignment to treatment and control group conducted?	
Does the study show baseline values of the outcomes of interest (as defined in the protocol) for beneficiaries and non‐beneficiaries?	
If baseline values of the outcome of interest are not available at baseline, does the study show baseline values of characteristics of beneficiaries and non‐beneficiaries that are not likely to be affected by the intervention?	
Are the mean values or the distributions of the covariates at baseline statistically different for beneficiaries and non‐beneficiaries (p<0.05)?	
If there are statistically significant differences between beneficiaries and non‐beneficiaries are these differences controlled for using covariate analysis in the impact evaluation?	
If baseline characteristics are not available, does the study qualitatively assess why beneficiaries are likely/unlikely to be a random draw of the population at baseline?	
**Confounding and selection bias**	
Does the study use a comparison/control group?	
Does the study include data on the outcomes of interest at baseline and end‐line (before and after the intervention)?	
Are the data on covariates collected at the baseline?	
Is difference‐in‐difference estimation used?	
If the study is quasi‐experimental and uses difference‐in‐difference estimation do the authors assess the parallel trends assumption?	
If the study does not use difference‐in‐difference, does the study control for baseline values of the outcome of interest (ANCOVA)	
If the study does not use difference‐in‐difference and does not control for baseline values of the outcome variable, does the study control for other covariates at baseline	
If the study does not use difference in differences estimation, is there any assessment of likely risk of bias from time invariant characteristics driving both participation and outcome?	
If the study does not use difference in difference estimation but does assess likely risk of bias from time invariant characteristics, are these time invariant characteristics likely to bias the impact estimates	
Does the study report the table with the results of the outcome equation (including covariates)?	
Where full results of the outcome equation are not reported, is it clear which covariates have been used?	
Are all relevant observable covariates (confounding variables) included in the outcome equation which might explain outcomes, if estimation does not use a statistical technique to control for selection bias (RCT, PSM, RDD, or IV)?	
**Attrition**	
For studies including baseline data, does the study report attrition (drop‐out) from the study?	
Is the attrition rate from the study below 10%?	
Does the study assess whether drop‐outs from the study are random draws from the sample (e.g. by examining correlation with determinants of outcomes, in both treatment comparison group)?	
**Spill overs and contamination**	
Spill‐overs: are comparisons sufficiently isolated from the intervention (eg participants and non‐participants are sufficiently geographically or socially separated) or are spill overs estimated by comparing non‐beneficiaries with access to the intervention to non‐beneficiaries without access to the intervention and/or through social network analysis?	
Spillovers; if spill overs are not estimated, is the study likely to overestimate or underestimate the impact of the program?	
Contamination: does the study assess whether the control group receives the intervention?	
Contamination: if the control group receives the intervention but for a shorter amount of time does the study assess the likelihood that the control group has received equal benefits as the treatment group	
Contamination: if the control group receives the intervention have they received the intervention sufficiently long to argue that they have benefited from the intervention	
**Other threats to validity**	
Does the evidence suggest analysis reporting biases are likely/unlikely to be serious? Analysis reporting biases include failure to report important treatment effects (possibly relating to intermediate outcomes), or justification for (uncommon) estimation methods, especially multivariate analysis for outcomes equations.	
**Hawthorne and John Hendry Effects**	
Do the authors argue convincingly that it is not likely that being monitored influences the behaviour of the beneficiaries and non‐beneficiaries in different ways?	
**Confidence Intervals**	
Does the study account for lack of independence between observations within assignment clusters if the outcome variables are clustered?	
Is the sample size likely to be sufficient to find significant effects of the intervention?	
Do the authors control for heteroscedasticity and/or use robust standard errors?	
Ask questions below only for studies that apply randomization	
Does the study apply randomized assignment?	
Does the study use a unit of allocation with a sufficiently large sample size to ensure equivalence between the treatment and the control group	
Ask questions below only for studies that apply regression discontinuity designs	
Is the allocation of the program based on a pre‐determined continuity on a continuous variable and blinded to the beneficiaries or if not blinded, individuals cannot reasonably affect the assignment variable in response to knowledge of the participation rule?	
Is the sample size immediately at both sides of the cut‐off point sufficiently large to equate groups on average?	
Is the mean of the covariates of individuals immediately at both sides of the cut‐off point statistically significantly different for beneficiaries and non‐beneficiaries?	
If there are statistically significant differences between beneficiaries and non‐beneficiaries are these differences controlled for using covariate analysis?	
Ask questions below only for studies that apply matching	
Quality of matching (PSM, covariate matching)	
Are beneficiaries and non‐beneficiaries matched on all relevant characteristics?	
Does the study report the results of the matching function (eg for PSM the logit function)?	
Does the study report the matching method?	
Does the study exclude observations outside the common support?	
Does the study use variables at follow‐up that can be affected by the intervention in the matching equation?	
Does the study report the mean or distribution for the covariates of the treatment and control groups after matching?	
Are these characteristics similar, based on tests for statistically significant differences (p>0.05)?	
**Ask questions below only for studies that apply instrumental variable estimation**	
Does the study describe clearly the instrumental variable(s)/identifier used?	
Are the results of the participation equation reported?	
Are the instruments jointly significant at the level of F ≥ 10? If an F test is not reported, does the author report and assess whether the R‐squared of the instrumenting equation is large enough for appropriate identification (R‐sq > 0.5)?	
Are the instruments individually significant (p≤0.05)?	
For IV, If more than one instrument is used in the procedure, does the study include and report an over identifying test (p≤0.05 is required to reject the null hypothesis)?	
Does the study qualitatively assess the erogeneity of the instrument/identifier (both externality as well as why the variable should not enter by itself in the outcome equation)?	
**Ask questions below only for studies with censored outcome variables**	
Do the authors use appropriate methods (e.g. Heckman selection models, tobit models, duration models) to account for the censoring of the data?	
For Heckman models; is there is a variable that is statistically significant in the first stage of the selection equation and excluded from the second stage	
**Risk of Selection Bias**	
**Risk of Performance Bias**	
**Risk of Outcome and Analysis Reporting Biases**	
**Risk of Other Biases**	

**Table 3:** Critical appraisal skills programme qualitative research checklist

**Criteria**	**Coding**
Screening Question: Is there a clear statement of study aims of the research?	Yes/Can't tell/No
Screening Question: Is a qualitative methodology appropriate?	Yes/Can't tell/No
Was the research design appropriate to address the aims of the research?	Yes/Can't tell/No
Was the recruitment strategy appropriate to address the aims of the research?	Yes/Can't tell/No
Were the data collected in a way that addressed the research question?	Yes/Can't tell/No
Has the relationship between researcher and participants been adequately considered?	Yes/Can't tell/No
Have ethical issues been taken into considerations?	Yes/Can't tell/No
Was the data analysis sufficiently rigorous?	Yes/Can't tell/No
Is there a clear statement of findings?	Yes/Can't tell/No

**Review Question 1:**

We will assess the risk of bias in included studies (RCT and nonrandomized) using risk of bias tool developed by 3ie (Hombrados & Waddington, 2012). The risk of bias tool will assess the likely risk of the following biases:

1) Selection bias based on quality of attribution methods (mechanism of assignment/identification) and assessment of group equivalence.
2) Performance bias based on the extent of spill overs to women in comparison groups.
3) Outcome analysis bias: Was the study free from selective outcome reporting?
4) Reporting bias: Was the study free from selective analysis reporting?
5) Other biases, including unit of analysis errors, coherence of results, retrospective baseline data collection, detection bias, and motivation and courtesy biases (Hawthorn effect; John Henri effect).

For details, please see risk of bias assessment tool (Table 2). We will assess risk of bias among these domains using the decision rules in the IDCG risk of bias tool (Hombrados & Waddington, 2012). We will assess the quality of a study by grouping them into high/medium/low risk of bias for each of the mentioned risk of bias categories:

Low risk of bias: appropriate and clearly described selection of participants, measurement of exposure and outcome variables, use of design, and use of analytical methods to control confounding, low risks of spillover or contamination, and, low risk of outcome and analysis reporting bias.

Medium risk of bias: inappropriate or unclear use of one of the following: (1) selection of participants, measurement of exposure and outcome variables, and use of design or analytical methods to control confounding; (2) assessment of risks of spillover or contamination; and (3) medium risk of outcome and analysis reporting bias.

High risk of bias: inappropriate use of two or more of the following: (1) selection of participants, measurement of exposure and outcome variables, and use of design or analytical methods to control confounding; (2) assessment of risks of spillover or contamination; and (3) high risk of outcome or analysis reporting bias.

Unclear risk of bias: unclear description of any of the following: (1) selection of participants, measurements of exposure and outcome, and use of study design or analytic methods to control for confounding, and (2) assessment of risks of spillover or contamination.

Two independent review authors will assess the quality of all studies using the risk of bias tool (Table 2). Each component will be first assigned an individual rating (Low = 1, Medium = 2, High = 3) indicating the overall potential for bias in each component. For example, an answer “Yes” will be coded L for a low risk of bias, an answer “No” will be coded as H for a high risk of bias and an answer “Unclear” will be coded as “M” for a medium risk of bias. Next, each study will be assigned a global rating. The criteria for an overall rating of a study will be as follows: Low risk of bias (no Weak ratings); Medium risk of bias (one Weak rating); High risk of bias (two or more Weak ratings). In the final step, the study will be graded as A (low risk of bias), or B (medium risk of bias), or C (high risk of bias).

Disagreements in the assessment of bias will be resolved through discussion between the two review authors. Where disagreements are not resolved, reasons for the discrepancy will be described. We will report risk of bias assessment for each included study.

**Review Question 2:**

We will critically appraise studies included to address question 2 using the Critical Appraisal Skills Programme Qualitative Research Checklist (CASP, 2006). We will assess the adequacy of stated aims, the data collection methods, the analysis, and the conclusion drawn. For details, please see Table 3.

For each item, two researchers will determine whether the study had adequately met the question criteria, and then score the question “yes” or “no” or “can't tell.” If the researchers disagree, they will discuss the item to reach a consensus. We will rate the studies that have scored 0–2 “no” or “can't tell” as studies with minimal risk of bias; studies that have scored 3–5 “no” or “can't tell” as studies with moderate risk of bias; and studies that have scored 6–9 “no” or “can't tell” as studies with high risk of bias.

Disagreements in the assessment of bias will be resolved through discussion between the two review authors. Where disagreements are not resolved, reasons for the discrepancy will be described. We will report quality assessment for each included study.

## Data collection and analysis

### Selection of studies

Citations will be stored in EndNote (bibliographic software). First, two review‐authors will screen the titles and abstracts obtained through the search strategy and identify potentially eligible studies independently. Next, using the EndNote comparison screening component, two authors will independently screen eligible studies where‐in the results of screened studies will be compared and differences in study inclusion will be reconciled after a discussion. If there is a disagreement or ambiguity about inclusion of a study based on the study title and the abstract/executive summaries, the full text of the article will be obtained to allow for further scrutiny of the eligibility of the study. Where there is a disagreement over the inclusion or exclusion of a study after a full text review, it will be resolved in discussion with a third reviewer and, where required, by the full review team.

### Data extraction and management

For review question 1, included studies will be screened in detail, and the data from each study will be extracted and entered into an Excel spreadsheet by one review author and, then, checked by a second review author. Similarly, for review question 2, data from included studies will be extracted and entered by one review author, and checked by a second review author.

Initially, two reviewers will independently double code data for the first 5 per cent of the identified studies. The data coding results will be compared for inter‐rater reliability. Any disagreements or inaccuracies will be checked and resolved by discussion. Reviewers will independently double code studies until a 100 per cent inter‐rater reliability is reached. When a 100 per cent inter‐rater reliability is reached, reviewers will switch to single coding. A full list of proposed draft data extraction fields is provided in the quantitative and qualitative data extraction forms (Appendix 3).

### Details of study coding categories

#### Review question 1

Data will be extracted on study characteristics (geographic context, i.e., region and country, and study population), study design, interventions, outcomes, and data for effect size calculations. A full list of proposed data extraction fields is provided in Data Extraction Form – I (Appendix 3). The data extraction form should be treated as a draft. The draft form will be developed during the data extraction phase of the review. If there is a disagreement over the inclusion or exclusion of data, it will be resolved after discussion with a third author.

#### Review Question 2

We will code studies on study characteristics (geographic context, i.e., region and country, and study population), study methodology, context, and thematic focus. We will extract data describing the views, experiences, and behaviours of women due to low or no access to road infrastructure, and transport & logistics services interventions. We will follow Munabi‐Babigumira et al. (2017) to extract authors' interpretations to represent the data of interest.

A full list of proposed data extraction fields is provided in Data Extraction Form – II (Appendix 3). The data extraction form should be treated as a draft. The draft form will be developed during the data extraction phase of the review. If there is disagreement over the inclusion or exclusion of data, it will be resolved after discussion with a third author.

### Statistical procedures and conventions

#### Measures of treatment effect

We will extract numerical data from each quantitative study to allow for the estimation of effect sizes, and associated standard errors with 95% confidence intervals.

For dichotomous outcomes, we will consider relative risk ratio effect measures:

Relative Risk (RR): the ratio of the treatment and the control proportions as follows:

R: Effect Size = $\frac{P_{T}}{P_{C}}$
*r_i_*: Effect Size for the *i^th^* study = $\frac{P_{T_{i}}}{P_{C_{i}}}$
$S_{\text{log}}\text{(}r_{i}\text{)}$: Standard Error of logarithm of effect size = $\sqrt{\left(\frac{\left(1-P_{T_{i}}\right)}{nT_{i}P_{T_{i}}}\right)\times \left(\frac{\left(1-P_{C_{i}}\right)}{nC_{i}P_{C_{i}}}\right)}$

The confidence intervals for relative risk will be computed on the logarithmic scale and transformed back to the original scale.

For continuous outcome: When the primary study reports means ($\bar{x}$) for the treatment and the control group, the effect size will be estimated using the mean difference (MD) or the standardized mean difference (SMD).

In mean difference, T denotes the treatment group and C denotes the control group with ‘*i*’ indexing the study,

MD: Effect Size = ${\bar{x}}_{T}-{\bar{x}}_{C}$
*MD_i_*: Effect Size for the *i^th^* study = ${\bar{x}}_{T_{i}}-{\bar{x}}_{C_{i}}$
$s_{i}^{2}$: Squared standard error of the effect size for the *i^th^* study =$S_{p_{i}}^{2}\left(\frac{1}{nT_{i}}+\frac{1}{nC_{i}}\right)$;
$S_{p_{i}}^{2}$ is the pooled sample variance defined as $S_{p_{i}}^{2}=\frac{\left(nT_{i}-1\right)S_{T_{i}}^{2}+(nC_{i}-1)S_{C_{i}}^{2}}{nT_{i}+nC_{i}-2}$, where $S_{T_{i}}^{2}$ and $S_{C_{i}}^{2}$ are the respective sample variance of the treatment and control group based on $nT_{i}$ and $nC_{i}$ observations.

When specific mean difference is standardized to obtain the scale free effect size called standardized mean difference (δ) defined as,

$\delta_{i}=\frac{MD_{i}}{S_{p_{i}}^{2}}$ along with the standard error SE $(\delta_{i})=\left(\frac{1}{nT_{i}}+\frac{1}{nC_{i}}\right)+\left(\frac{\delta_{i}^{2}}{2\left(nT_{i}+nC_{i}\right)}\right)$

For count outcomes: when the outcome variable is a count, the effect size will be based on rate ratio. Assuming that for the i^th^ study, observed counts for treatment and control group is $nT_{i}$ and $nC_{i}$ respectively and the corresponding rates being $\lambda T_{i}$ and $\lambda C_{i}$, then rate ratio is defined as $\left(RR_{i}=\frac{\lambda T_{i}}{\lambda T_{i}}\right)$. Here, the logarithm of the risk ratio works as the effect size i.e.

$\gamma_{i}=\ln\left(\frac{\lambda T_{i}}{\lambda T_{i}}\right)$ with $\text{SE}(\gamma_{i})=\sqrt{\left(\frac{1}{nT_{i}}+\frac{1}{nC_{i}}\right)}$

If a study contains a zero count in either the control group or the treatment group, the analysis is modified by adding a “continuity correction” of 0.5 to the counts from both groups.

For publication bias correction, we will use Copas model. The Copas selection model comprises of two components: 1) a random effects model for the outcome; 2) a selection model giving the probability that a study is observed or published. A correlation parameter between these two components models the extent of publication bias. An iterative procedure is employed where the selection model is refitted with increasing the probability of publishing while monitoring the selection bias. When the test for residual selection bias is no longer significant, the Copas selection model stops and returns an adjusted estimate of the underlying treatment effect. The Copas selection model is based on the maximization of a complex likelihood function, and estimation problems cannot be ruled out from sometimes affecting the estimation process.

### Unit of analysis issues

We will assess studies for unit of analysis errors, where the unit of the treatment is different from the unit of analysis. For those studies that have used a clustered design and that have not adjusted for clustering when reporting their data, we will determine design effect using the interclass correlation coefficient (ICC) of the study, if available; and the average cluster size. Where ICCs are not reported we will use ICC values from similar, published cluster randomized trials. The outcomes will then be corrected using the design effect. Where a study will include multiple intervention groups, we will combine data from all relevant intervention groups of the study into a single group and separately combine data from all relevant control group.

### Publication bias assessment

We will examine funnel plots and cumulative meta‐analysis to assess the potential for publication bias in included studies. Provided a sufficient number of studies are available, we will apply Copas model for correcting publication bias using R software version 3.1.2.

### Dealing with missing data

As far as possible, we will analyze data on an intention‐to‐treat basis. Where data are missing, we will try to obtain the data from the authors. If we are unable to obtain missing data, we will extract or impute the effect size based on commonly reported statistics, such as, the t or F statistics, or p or z values using David Wilson's practical meta‐analysis effect size calculator.

## Data synthesis

We will conduct an integrated review using “mixed research synthesis” approach (Sandelowski et al., 2006) to synthesize research evidence. We will synthesize quantitative evidence by conducting meta‐analysis of numerical data from quantitative trials. We will do meta‐synthesis of the narrative data from qualitative research.

We will integrate the findings from the qualitative synthesis with those from the meta‐analysis and develop a series of matrices for assessing how gender‐responsive road infrastructure, and transport & logistics interventions can impact women's empowerment.

For integrating the quantitative and the qualitative data synthesis, this review will sequence aggregative (quantitative) synthesis using meta‐analysis, followed by configuring (qualitative) synthesis using meta‐synthesis approach. The qualitative synthesis will be used to investigate more in depth aspects that the findings of the quantitative synthesis may not be able to provide a good answer.

**Review question 1:**

We will group studies by intervention category first, then by study design, and the data within these groupings will be presented by outcome domain. We will report meta‐analysis results of randomized control trials and non‐randomized studies separately.

The calculations for all of the effect sizes will be done using the statistical software R version 3.1.2. There will be a subset for each study indexed by a reference identifier and by the first author and year. For dichotomous outcome results, we will use the risk difference risk ratio (RR) with 95% confidence intervals (CIs). For continuous outcomes, we will use standardized mean differences (SMDs) with 95% CI. When the outcome variable is a count, the effect size will be based on the rate ratio.

After the corresponding effect size and its standard error from the studies are obtained, we would apply inferential procedures to gain more insights about the population level effect size or the average effect size. Given the diverse sets of interventions and outcomes, a meta‐analysis of effect sizes will be conducted using a random‐effect model. The degree of between‐study heterogeneity will be assessed using Chi² and I² statistics. If there is sufficient data available, we will attempt to fit random‐effects meta‐regression model to determine whether study‐level variables like the place of study, the type of intervention, the duration of follow‐up, and the study setting can explain the heterogeneity in the effect sizes.

If there is an extreme heterogeneity, such that the aggregation of effect sizes in a meta‐analysis or meta‐analysis within sub groups is not possible; we will present the effect sizes of included studies graphically using forest plots together with statistical power analysis, and a narrative synthesis of the data to be presented according to the ESRC guidance (Popay 2006). Where outcomes are considered sufficiently similar for meta‐analysis but not all the available data is amenable to calculating a standardized effect size estimate due to poor reporting, the available data will be meta‐analysed, and the complete data for that outcome will be narratively synthesized.

### Sensitivity analysis

Before making decisions about which studies should be included in the final syntheses, a sensitivity analysis will be conducted to examine variation in reported effects by study characteristics, provided a sufficient number of studies are available. The key study characteristics used for sensitivity analysis will be the following: study design (RCT vs non‐randomized), geographic region (LIC vs LMIC), and the study quality.

### Subgroup analysis

Due to the broad scope of this review, the identified studies will display extreme variation in the study methods used, the interventions being assessed, the study populations, and the potential range of outcome scale being assessed. Thus, we plan to undertake several subgroup analyses provided a sufficient number of studies are available:

‐ Intervention category: Transport access to basic utility services and markets; Access to labour markets; and, Gender capacity building in transport sector;
‐ Study design;
‐ Low‐income countries versus middle‐income countries;

### Assessment of heterogeneity

We expect the variations in the study findings to crop up due to various sources of heterogeneity (for example, study population, region, and macroeconomic environment of the country in which the study was conducted). We will assess the heterogeneity of studies by inspection of the forest plot and, in particular, the confidence intervals of the individual studies; statistical tests of heterogeneity will be undertaken using the Chi² test, with significance defined as a P value of < 0.05; and the I² statistic. The standardized effects will be plotted on a forest plot and, provided there is high level of heterogeneity (I² > 75%), meta‐analysis will not be performed and efforts will be made for investigating the sources of heterogeneity.

Finally, we will use GRADE to present the findings from quantitative research.

**Review question 2:**

We will use meta‐synthesis method to examine women's experiences and views of constraints (or drive) that fallout as a result of gender‐responsive road infrastructure related transport and logistics interventions. Because we are looking for contextual dimensions to describe factors, in different studies but interrelated interventions and context, meta‐synthesis approach is the most appropriate to answer our research question. According to Walsh & Downe (2005), meta‐synthesis attempts to integrate results from a number of different but interrelated qualitative research.

In the initial examination of the synthesis stage, we will attempt to cluster studies of the same or closely related methodological approaches in a single meta‐synthetic set (Campbell et al., 2003). During this stage, we will code included studies on study characteristics, study methodology, epistemological perspective, context, and theoretical framing.

In the next stage, we will endeavor characterization of included studies, and then develop middle‐range and generalizing theories about their findings. We will use analytical technique suggested by Walsh et al. (2005, p. 208) for the synthesis of included studies:

1) For all included studies, we will identify and tabulate the original author's understanding of key phrases, ideas, concepts, and relations from the narrative text in each study. Studies will be summarized to draw inferred themes and concepts. The rational and consequences of the decisions will be explained.
2) Thematic coding: Next, the findings of the included studies will be thematically analyzed. A line by line coding of findings will be organized into “descriptive” themes.
3) Summaries of the research findings between the studies will be compared in order to identify similarities and differences of categories/codes/themes.
4) In the next phase, we will translate categories/codes/themes between studies by using higher abstract level “analytical” themes. Finally, we will synthesize the findings into explanatory theories. During this phase, we will encounter deviant findings, which may be refutations and overlap translations. This deviant data may be the raw material of another perspective or a different thought process.

In some cases, it is possible that the published studies do not seem to be adequate for the conclusions drawn using them. In such cases, we will seek other reports of the same publication.

While analyzing themes, it is possible to force a fit in the interest of illustrating homogeneity across the themes/codes/categories. We will try to maintain the process of preserving the meaning from the original text as far as possible through openness and transparency by explaining the rational and the consequences of decisions.

Finally, we will use GRADE‐CerQual to present the findings from included studies.

### Integrating findings from quantitative synthesis of effectiveness with qualitative synthesis

After each set of qualitative and quantitative findings has been separately synthesized, we will present a mixed research synthesis of the findings as stated in the research work of Sandelowski, Viols, & Barrows (2006). We will develop a series of matrices to identify the features of interventions and context (from women's experiences/perspectives) that empower and/or constrain the effectiveness of road infrastructure, and transport & logistics interventions towards women.

## ACKNOWLEDGEMENTS

We wish to thank the following Review Advisory Group members whose expertise made this document possible: Priyanthi Fernando from IWRAW Asia Pacific and Elena Bardasi from Independent Evaluation Group, World Bank.

## Review authors

**Lead review author:** The lead author is the person who develops and co‐ordinates the review team, discusses and assigns roles for individual members of the review team, liaises with the editorial base and takes responsibility for the on‐going updates of the review.

**Table:** 

Name:	Manisha Gupta
Title:	Principal Investigator
Affiliation:	Independent Researcher
Address:	
City, State, Province or County:	Delhi
Post code:	110096
Country:	India
Phone:	
Email:	manischa.gupta@gmail.com
**Co‐author(s):** (There should be at least one co‐author)	
Name:	**Souvik Bandhyopadhyaya**
Title:	Associate Professor
Affiliation:	Indian Institute of Public Health, Hyderabad‐ Public Health Foundation of India
Address:	
City, State, Province or County:	Hyderabad, Telangana
Post code:	500033
Country:	India
Phone:	
Email:	bansauvik@gmail.com
Name:	**Dr Meerambika Mahapatro**
Title:	Associate Professor
Affiliation:	National Institute of Health and Family Welfare (NIHFW)
Address:	
City, State, Province or County:	Delhi
Postal Code:	110096
Country:	India
Phone:	
Email:	Meerambika.mahapatro@gmail.com
Name:	**Ms. Shreya Jha**
Title:	Qualitative Researcher
Affiliation:	PhD candidate at Bath University
Address:	
City, State, Province or County:	Noida, Uttar Pradesh
Postal Code:	
Country:	India
Phone:	
Email:	Shreyajha00@gmail.com
Name:	**Poh Chua**
Title:	Deputy Librarian
Affiliation:	The Royal Children's Hospital
Address:	
City, State, Province or County:	Parkville Victoria
Postal Code:	3052
Country:	Australia
Phone:	
Email:	Poh.chua@rch.org.au

## Roles and responsibilities

**Table:** 

**Name**	**Subject**	**Role in the Review**	**Responsibility**
Ms. Manisha Gupta	Content (Transport) and Systematic review methods	Principal investigator/lead co‐author	Lead the review in screening, data extraction, appraisal, synthesis, and report writing; coordination with IDCG and advisory group
Dr Sauvik Bandhyopadhyaya	Statistical Analysis	Meta‐analysis on R software expert and co‐author	Responsible for quantitative studies meta‐analysis on R‐software
Dr Meerambika Mahapatro	Content (Women empowerment and thematic analysis)	Thematic analysis and women empowerment Expert and co‐author	Responsible for methodological aspects of qualitative synthesis and women empowerment
Ms. Shreya Jha	Content (Women empowerment and thematic analysis)	Qualitative analysis researcher and co‐author	Responsible for screening, qualitative data extraction, appraisal, and thematic analysis
Poh Chua	Information retrieval	Information/search Specialist	Responsible for developing the search strategy and running the search in databases

## Sources of support

At present we do not have any funding support. We will explore opportunities when available.

## Declarations of interest

None.

## Preliminary timeframe

Approximate date for submission of the systematic review: 31 July 2019

## Plans for updating the review

The lead co‐author/principal investigator will be responsible for updating the review during a period of two years.

## AUTHOR DECLARATION

### Authors' responsibilities

By completing this form, you accept responsibility for preparing, maintaining and updating the review in accordance with Campbell Collaboration policy. The Campbell Collaboration will provide as much support as possible to assist with the preparation of the review.

A draft review must be submitted to the relevant Coordinating Group within two years of protocol publication. If drafts are not submitted before the agreed deadlines, or if we are unable to contact you for an extended period, the relevant Coordinating Group has the right to de‐register the title or transfer the title to alternative authors. The Coordinating Group also has the right to de‐register or transfer the title if it does not meet the standards of the Coordinating Group and/or the Campbell Collaboration.

You accept responsibility for maintaining the review in light of new evidence, comments and criticisms, and other developments, and updating the review at least once every five years, or, if requested, transferring responsibility for maintaining the review to others as agreed with the Coordinating Group.

### Publication in the Campbell Library

The support of the Coordinating Group in preparing your review is conditional upon your agreement to publish the protocol, finished review, and subsequent updates in the Campbell Library. The Campbell Collaboration places no restrictions on publication of the findings of a Campbell systematic review in a more abbreviated form as a journal article either before or after the publication of the monograph version in Campbell Systematic Reviews. Some journals, however, have restrictions that preclude publication of findings that have been, or will be, reported elsewhere and authors considering publication in such a journal should be aware of possible conflict with publication of the monograph version in Campbell Systematic Reviews. Publication in a journal after publication or in press status in Campbell Systematic Reviews should acknowledge the Campbell version and include a citation to it. Note that systematic reviews published in Campbell Systematic Reviews and co‐registered with the Cochrane Collaboration may have additional requirements or restrictions for co‐publication. Review authors accept responsibility for meeting any co‐publication requirements.

**I understand the commitment required to undertake a Campbell review, and agree to publish in the Campbell Library. Signed on behalf of the authors**:

**Form completed by: Manisha Gupta, 26 July 2018**

## APPENDIX 2

### DRAFT SEARCH STRATEGY

Ovid‐Medline database search run yielded 10, 7210 searches on June 18, 2018.

**P ‐ Population terms:**

1. (wom#n* or female* or girl* or schoolgirl* or school‐girl* or wife or wives or mother* or gender* or sex‐disaggregat* or businesswom#n or business‐wom#n) AND (employment or employab* or employer* or employee* or work or workplace or work‐place or work‐force or workforce or job* or vocation* or career* or occupation* or livelihood* or part‐time or casual or informal* or wage* or labo?r‐market* or school‐to‐work or school or schools
2. (”transitioning econom*“or” emerging countr*“or develop* count*” or “developing countries” or “transitional countr*”)
3. “(lmic or lmics or third world or lami countr*)”
4. ((“developing or less* developed or under developed or underdeveloped or middle income or low* income) adj (economy or economies”))
5. ((developing or less* developed or under developed or underdeveloped or middle income or low* income or underserved or under served or deprived or poor*) adj (countr* or nation? or population? or world or state*))
6. Afghanistan or Albania or Algeria or Angola or Argentina or Armenia or Armenian or Azerbaijan or Bangladesh or Benin or Byelarus or Byelorussian or Belarus or Belorussian or Belorussia or Belize or Bhutan or Bolivia or Bosnia or Herzegovina or Hercegovina or Botswana or Brazil or Bulgaria or Burkina Faso or Burkina Fasso or Upper Volta or Burundi or Urundi or Cambodia or Khmer Republic or Kampuchea or Cameroon or Cameroons or Cameron or Camerons or Cape Verde or Central African Republic or Chad or China or Colombia or Comoros or Comoro Islands or Comores or Mayotte or Congo or Zaire or Costa Rica or Cote d'Ivoire or Ivory Coast or Croatia or Cuba or Djibouti or French Somaliland or Dominica or Dominican Republic or East Timor or East Timur or Timor Leste or Ecuador or Egypt or United Arab Republic or El Salvador or Eritrea or Ethiopia or Fiji or Gabon or Gabonese Republic or Gambia or Gaza or Georgia Republic or Georgian Republic or Ghana or Gold Coast or Grenada or Guatemala or Guinea or Guiana or Guyana or Haiti or Honduras or Hungary or India or Maldives or Indonesia or Iran or Iraq or Jamaica or Jordan or Kazakhstan or Kazakh or Kenya or Kiribati or Korea or Kosovo or Kyrgyzstan or Kirghizia or Kyrgyz Republic or Kirghiz or Kirgizstan or Lao PDR or Laos or Lebanon or Lesotho or Basutoland or Liberia or Libya or Macedonia or Madagascar or Malagasy Republic or Malaysia or Malaya or Malay or Sabah or Sarawak or Malawi or Mali or Marshall Islands or Mauritania or Mauritius or Agalega Islands or Mexico or Micronesia or Middle East or Moldova or Moldovia or Moldovian or Mongolia or Montenegro or Morocco or Ifni or Mozambique or Myanmar or Myanma or Burma or Namibia or Nepal or Netherlands Antilles or New Caledonia or Nicaragua or Niger or Nigeria or Pakistan or Palau or Palestine or Panama or Paraguay or Peru or Philippines or Philipines or Phillipines or Phillippines or Papua New Guinea or Romania or Rumania or Roumania or Rwanda or Ruanda or Saint Lucia or St Lucia or Saint Vincent or St Vincent or Grenadines or Samoa or Samoan Islands or Navigator Island or Navigator Islands or Sao Tome or Senegal or Serbia or Montenegro or Seychelles or Sierra Leone or Sri Lanka or Solomon Islands or Somalia or Sudan or Suriname or Surinam or Swaziland or South Africa or Syria or Tajikistan or Tadzhikistan or Tadjikistan or Tadzhik or Tanzania or Thailand or Togo or Togolese Republic or Tonga or Tunisia or Turkey or Turkmenistan or Turkmen or Uganda or Ukraine or Uzbekistan or Uzbek or Vanuatu or New Hebrides or Venezuela or Vietnam or Viet Nam or West Bank or Yemen or Yugoslavia or Zambia or Zimbabwe).ti,ab,hw,ct.
7. (Africa or Asia or Caribbean or West Indies or South America or Latin America or Central America)
8. **1 AND (2 or 3 or 4 or 5 or 6 or 7)**

**I – Interventions terms**

***Logistics terms***

9. tariff* or non‐tariff* or right* or oblig* or (clearance adj system*) or (border adj requirement*) or (custom* adj process*)

10. border* or (border adj crossing*) or (border adj custom*) or (transport adj hub*) or storage* or (cross adj border*)

11. skill* or upskill* or up‐skill* or train* or retrain* or mentor* or value* or prestige or expertise or professional* or qualifi* or educat* or coaching or leadership or entrepreneur* or (human adj capital) or (business* adj2 (manag* or support*)) or (capacity adj2 (build* or acqui*)) or empower* or segregat* or stratif* or constrain* or barrier* or facilitate* or logistic*

**12. or/9‐11**

***Road engineering terms***

13. road* or pavement* or (two‐way‐traffic) or straightway* or dual‐carriageway* or motorway* or motor‐way* or traffic‐light* or highway*

14. transport* and (construct* or contract* or consultation* or appraisal or maintenance or planning or infrastructure* or intervention* or program* or service* or cost* or activit*)

***Intermediate Transport terms***

***Public Transport terms***

15. (push‐bike* or bicycle* or cyclist* or cycling or bike* or motor‐cycl* or moto‐cycl* or motorcycl* or motocycl* or motorbike* or motor‐bike* or scooter* or moped* or pedestrian*) and (cross* or lane* or facilit* or way* or walkway* or walk‐way* or path* or foot* or sidewalk* or side‐walk* or platform* or overpass* or over‐pass* or underpass* or under‐pass* or boulevard* or track* or route* or shoulder* or program*)

16. bus or buses or taxi* or cab or cabs or minibus* or ferry or ferries or train or trains or rail or railways (public adj transport* or public service vehicle*)

17. IMT or (intermediate adj3 transport*) or (finance* adj3 cost*) or credit or scheme* or fare or fares

18. or/13‐17

19. (push‐bike* or bicycle* or cyclist* or cycling or bike* or scooter* or moped* or pedestrian*)

20. Commute* or travel* or walk* or drive*

21. (safety or security or sensitivity)

**S ‐ Study type**

22. Controlled AND (trial or trials or study or studies or experiment)

23. Randomized controlled trials.

24. (Non‐randomi* AND (trial or trials or study or studies or experiment)

25. Uncontrolled (random allocation / or clinical trial/ or single‐blind method/ or double‐blind method/ or control groups/)

26. Retrospective study/

27. Impact evaluation studies/

28. Prospective study/

29. Analytical cross‐sectional comparative studies/

30. Descriptive cross‐sectional study/

31. Intervention studies/

32. quasi‐experiment*

33. ((pre test or pretest or (posttest or post test))

34. trial.ti.

35. (time adj series)

36. ((evaluat* or intervention or interventional) adj8 (control or controlled or study or program* or comparison or” before and after “or comparative))

37. ((intervention or interventional) adj8 (effect* or evaluat* or outcome*))

38. (controlled before or ‘before and after stud$’ or follow up assessment)

39. caseseries/

40. intervention adj1 group$

41. or/22‐40

**42. 8 AND 12 AND 41**

**43 8 AND (18 OR 19 OR 20 OR 21) AND 41**

**44. 42 OR 43**

**45. limit 44 to yr=“1990‐2018”**

## APPENDIX 3

### DATA EXTRACTION FORM – I QUANTITATIVE STUDIES

**Table:**

**STUDY**	
Study Id	
Coder Initials	
Author (s)	
Funder	
Publication date	
Country	
Publication type	Book
	Peer‐reviewed journal
	Book chapter
	Dissertation/thesis
	Unpublished report

**STUDY DESIGN**				
RCT	Non randomized study			
Non randomized	CBA	Uncontrolled before and after	ITS	Observational study
Sample size				
Comparison group				
Type of sampling	Random	Purposive	Convenience	Cannot tell
Did researchers use baseline data	Yes		No	
If Yes, were there differences	No	Minor	Major	Cannot tell

**INTERVENTION**	
Intervention category	
Describe the intervention	
Duration of the intervention	
What information is provided about what the comparison/control group received	

**RESULTS**		
Are the main findings of the study clearly described? (Simple outcome data including denominators and numerators should be reported for all major findings so that the reader can check the major analysis and conclusions)	Yes	No
Does the study report secondary outcomes	Yes	No
Does the study provide cost data		
Does the study report baseline data		

**OUTCOMES**				
Source of information	Survey	Records	Interviews	Focus groups
Type of information	Quantitative	Qualitative		
Quantitative outcome category	Primary	Secondary		
Outcome names/definition				
Measure/indicator of outcome				
Were there any differences in measurement of outcomes between the group participants and the comparison	Yes	No	Cannot tell	

**EFFECT SIZE**		
For quantitative studies		
Outcome Category	Primary	Secondary
Outcome Name		
Intervention group sample size		
Comparison group sample size		
For Continuous Measures:		
Intervention group mean		
Comparison group mean		
Are means reported above adjusted	Yes	No
Intervention group standard deviation		
Comparison group standard deviation		
Intervention group standard error		
Comparison group standard error		
T‐value from an independent t‐test		
For Dichotomous Measures:		
Intervention group number of participants who experienced a change		
Comparison group number of participants who experienced a change		
Intervention group proportion of participants who experienced a change		
Comparison group proportion of participants who experienced a change		
Are the proportions above adjusted for pretest variables	Yes	No
Logged odds ratio		
Standard error of logged odds ratio		
Logged odds ratio adjusted (e.g. from a logistic regression analysis with other independent variables)	Yes	No
Chi‐square value with df=1		
	
Correlation coefficient		
For Hand Calculated Data:		
Hand calculated d‐type effect size		
Hand calculated standard error of the d‐type effect size		
Hand calculated odds ratio effect size		
Hand calculated odds ratio standard error		

### DATA EXTRACTION FORM – II QUALITATIVE STUDIES

**Table:**

**Study id**	
**Code's id**	
**Name of the study under research question 2**	
**Date coded**	
**STUDY CHARACTERISTICS**	
Name of the country	
Name of the region	
Target Group: (1) Women only, (2) Women and Men	
Rural or Urban Area: (1) Rural, (2) Urban, (3) Both Rural and Urban	
**INTERVENTION**	
Intervention category	
Describe the intervention	
Duration of the intervention	
What information is provided about what the comparison/control group received	
Descriptive details about what is delivered to participants:	
Descriptive details about who delivers the Intervention (profession training level, number of staff etc.)	
Descriptive details about duration/frequency/intensity of training:	
Sector:	
Target Group: (1) Women only, (2) Women and Men	
Rural or Urban Area: (1) Rural, (2) Urban, (3) Both Rural and Urban	
**STUDY METHODOLOGY**	
Data Collection Type: 1) Focus group 2) In‐depth interviews 3) Ethnographies 4) Participatory research 5) Other	
Sampling: 1) Random 2) Purposive	
Sample size	
Description of the sampling procedure	
Description of Themes	
Description of Analytical Themes	
